# Screening for 22q11 deletion syndrome among patients with congenital heart defects

**DOI:** 10.1590/1516-3180.2014.1322655

**Published:** 2014-04-01

**Authors:** Rafael Fabiano Machado Rosa, Rosana Cardoso Manique Rosa, Patrícia Trevisan, Carla Graziadio, Marileila Varella-Garcia, Giorgio Adriano Paskulin, Paulo Ricardo Gazzola Zen

**Affiliations:** I PhD. Professor of Postgraduate Program on Pathology, Universidade Federal de Ciências da Saúde de Porto Alegre (UFCSPA), and Clinical Geneticist, Universidade Federal de Ciências da Saúde de Porto Alegre (UFCSPA), Complexo Hospitalar Santa Casa de Porto Alegre (CHSCPA) and Hospital Materno Infantil Presidente Vargas (HMIPV), Porto Alegre, Rio Grande do Sul, Brazil; II MD. Postgraduate Student, Postgraduate Program on Pathology, Universidade Federal de Ciências da Saúde de Porto Alegre (UFCSPA), Porto Alegre, Rio Grande do Sul, Brazil; III MD. Postgraduate Student, Postgraduate Program on Pathology and Assistant Professor of Clinical Genetics, Universidade Federal de Ciências da Saúde de Porto Alegre (UFCSPA); and Clinical Geneticist, Universidade Federal de Ciências da Saúde de Porto Alegre (UFCSPA) and Complexo Hospitalar Santa Casa de Porto Alegre (CHSCPA), Porto Alegre, Rio Grande do Sul, Brazil; IV PhD. Professor of Medical Oncology and Director of the Cancer Center Cytogenetics Core, University of Colorado Denver (UCD), Aurora, Colorado, United States; PhD. Associate Professor of Clinical Genetics and Coordinator of the Postgraduate Program on Pathology, Universidade Federal de Ciências da Saúde de Porto Alegre (UFCSPA); and Clinical Geneticist, Universidade Federal de Ciências da Saúde de Porto Alegre (UFCSPA) and Complexo Hospitalar Santa Casa de Porto Alegre (CHSCPA), Porto Alegre, Rio Grande do Sul, Brazil; PhD. Adjunct Professor of Clinical Genetics and Professor of the Graduate Program in Pathology, Universidade Federal de Ciências da Saúde de Porto Alegre (UFCSPA); and Clinical Geneticist, Universidade Federal de Ciências da Saúde de Porto Alegre (UFCSPA) and Complexo Hospitalar Santa Casa de Porto Alegre (CHSCPA), Porto Alegre, Rio Grande do Sul, Brazil

The 22q11 deletion syndrome (22q11DS), or velocardiofacial/DiGeorge syndrome, is considered to be the second most known genetic cause of congenital heart disease (CHD).^1^ Our aim was to evaluate the effectiveness of different screening methods for 22q11DS in patients with CHD. Our study evaluated a consecutive sample of patients with CHD hospitalized for the first time in a pediatric and cardiac intensive care unit of a referral hospital in southern Brazil. All of them underwent the examination through fluorescent *in situ* hybridization for 22q11DS. These patients were part of the study by Rosa et al.^2^ CHDs were classified by a cardiologist as conotruncal or non-conotruncal. We excluded patients with other chromosomal abnormalities. Three different approaches composed the screening:

(1) Testing suggested by Tobias et al.^3^ The clinical findings are divided into three categories:

1. conotruncal CHD;
2. abnormalities common in 22q11DS, such as hypocalcemia and non-conotruncal CHD; and
3. abnormalities less common in 22q11DS, such as short stature and hypotonia. Patients that have an alteration in group A, two findings in group B, or one finding in group B plus one in group C are tested;

(2) Testing suggested by the American Heart Association (AHA) Congenital Cardiac Defects Committee,^4^ which consists of testing all newborns/infants with interrupted aortic arch (IAA), truncus arteriosus (TA), tetralogy of Fallot (TOF), ventricular septal defect (VSD) (perimembranous conoseptal hypoplasia or malalignment) with aortic arch anomaly (AAA), AAA alone and discontinuous branch pulmonary arteries. The screening also includes any newborn/infant/child with CHD associated with another feature of 22q11DS (such as hypocalcemia, facial dysmorphia and palate abnormality); newborns/infants with VSD, and any child/adolescent/adult with TOF, TA, IAA, VSD or AAA who has one other feature of 22q11DS; 

(3) Testing performed at some centers,^5^ where only patients with conotruncal heart defects are tested.

**Figure 1:**
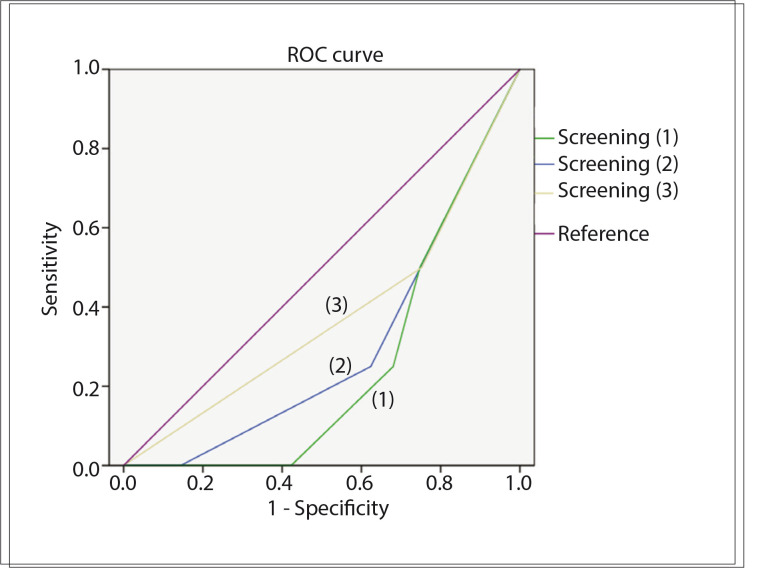
Receiver operating characteristic (ROC) curves presented by the different screening criteria

For all these approaches, we calculated the sensitivity, specificity, positive predictive value (PPV), negative predictive value (NPV) and receiver operating characteristic (ROC) curves. The significance level used was 5% (P ≤ 0.05). The total sample consisted of 170 patients (93 males), with ages ranging from 1 to 4934 days (mean of 847.7 days, standard deviation of 1225.1). 22q11DS was identified in four patients (2.4%): two newborns with TOF, one newborn with VSD associated with AAA, and one adolescent with atrial septal defect. One hundred and eleven patients (65%) met screening criterion 1, with sensitivity 100%, specificity 36%, PPV 3.6% and NPV 100%. Criterion 2 was met by 76 (44.7%) patients, with sensitivity 75%, specificity 56%, PPV 3.9% and NPV 98.9%. Forty-five patients (26.5%) had conotruncal heart defects and fulfilled criterion 3, with sensitivity 50%, specificity 74%, PPV 4.4% and NPV 98.4%. The ROC curves are shown in Figure 1. All the areas under the ROC curves were less than 0.5 [criterion 1: 0.247 (P = 0.083); criterion 2: 0.295 (P = 0.161); and criterion 3: 0.374 (P = 0.388)].

Method 1 showed the highest sensitivity (100%). On the other hand, method 3 identified only half of the cases. However, it is noteworthy that the PPV was very low in all the approaches, i.e. many patients needed to be screened in order to diagnose the individuals with 22q11DS, and in some circumstances not all of them were diagnosed. The analysis on the ROC curves also showed that none of the criteria used presented satisfactory performance, since all of them presented areas under the ROC curve of less than 0.5. Thus, new approaches are still needed, especially with the aim of reducing the costs involved in screening. It is possible that new and cheaper technologies such as multiplex ligation-dependent probe amplification may become applicable as screening methods.
